# The impact of household wealth gap on individual’s mental health

**DOI:** 10.1186/s12889-023-16871-6

**Published:** 2023-10-06

**Authors:** Rui Zhang, Dawei Feng, Jiahui Xia, Yao Wang

**Affiliations:** 1 School of Economics, Xihua University, Chengdu, China; 2 School of Applied Economics (School of Digital Economics), Jiangxi University of Finance and Economics, Nanchang, 330013 China; 3 School of Management, Jinan University, Guangzhou, China; 4 School of Economics, Jinan University, Guangzhou, China

**Keywords:** Wealth gap, Mental health, Health insurance, Neighborhood relations, Medium- to long-term effects

## Abstract

**Background:**

Improving the individual’s mental health is important for sustainable economic and social development. Although some studies found that household wealth gap may affect individuals’ mental health, few studies have clarified the causal relationship between household wealth gap between mental health in China. This study examines the impact of the household wealth gap on individuals’ mental health using data from the 2012–2018 China Family Panel Survey.

**Methods:**

This study first used the two-way fixed effects model to investigate the impact of household wealth gap on individuals’ mental health. Considering the endogeneity, the two-stage least square and propensity score matching were employed to examine the impact of household wealth inequality on individuals’ mental health.

**Results:**

The results show that the household wealth gap has negative impact on individuals’ mental health. A series of robustness tests support this conclusion. The results of heterogeneity analysis show that the impact of household wealth gap on mental health is more pronounced among middle-aged and elderly individuals, residents with lower education levels, and rural residents. The results of the mechanism analysis suggest that the household wealth gap may affect individuals’ mental health by influencing the individual’s health insurance investment and neighborhood relations. In addition, the household wealth gap not only significantly negatively affects individuals’ mental health in the short term but also in the medium- to long-term.

**Conclusion:**

These findings suggest that the government should take various measures to narrow the wealth inequality between families, which may effectively improve the mental health of residents.

## Introduction

The wealth gap is widening in countries worldwide due to globalization, technological progress, housing prices, and various policies and external factors such as distribution, welfare, and taxation. China was once the country with the most equalized income distribution globally. With the rapid economic development, although the major population has become more wealthy, the wealth gap among households has widened at the same time [1, 2]. Credit Suisse’s Global Wealth Report 2021 suggested that the richest 10% of the world’s population hold about 82% of the world’s wealth at the end of 2020, indicating that the wealth gap is relatively large worldwide [3]. At the same time, the report also revealed that the global wealth gap tends to widen further in 2020 under the COVID epidemic, with the richest 1% of the world’s population, on average, seeing their wealth share rise by 1.1%. Among them, the wealth of the richest 1% in China rose by 1.6%. In addition, previous study shows that the richest 10% of China’s population held 67% of the national wealth in 2015, more than most of the European developed countries such as the UK and France [4].Evidence shows that the wealth gap among Chinese residents is already high and tends to widen further.

Undeniably, a certain wealth gap can encourage individuals and enterprises to innovate and promote economic and social development. However, a large wealth gap can substantially negatively impact society and families. It may cause severe class antagonism and social division, lead to conflicts and wars. A large number of studies have focused on the measurement of the wealth gap [5, 6] and the causes of the wealth gap [1, 7, 8]. Few studies have examined the impact of the wealth gap on individuals, and even fewer studies have examined the impact on the mental health of Chinese residents.

The wealth gap can affect the individual’s health through different channels. It has been shown that individuals tend to compare upwards, with people tending to compare themselves with those in the reference group who are wealthier, causing the feeling of relative deprivation [9]. It has long been recognized that the income gap can negatively impact individual’s health [10]. The relative income hypothesis suggests that relative deprivation experienced by individuals can significantly negatively affect the health status of the population [11].

The wealth gap study, however, is more valuable than the study of the income gap [12]. Regarding theoretical value, whether inequality in economic resource allocation improves or harms individuals’ health is a critical question in social science research. The essence behind the previous question is based on wealth distribution rather than income distribution. In reality, the wealth gap between households in China is becoming more and more prominent. The wealth gap is harder to control than the income gap since it may exist throughout different generations, indicating that the impact of household wealth gap on individuals’ health may be more profound. Focusing solely on the income gap may underestimate the potentially substantial impact [12]. Few previous studies examined the causal relationship between the household wealth gap and the individual’s mental health. As the household wealth gap in China widens further, this study examines both the short- and medium- to the long-term impact of the household wealth gap on individuals’ mental health in China.

The contributions of this study are reflected in the following aspects: First, most previous studies only examined the impact of the household wealth gap on individuals’ health in developed countries. Different from those studies, this study examines the short- and medium- to long-term impact of the household wealth gap on the mental health of Chinese residents from theoretical and empirical perspectives. Second, this study examines the impact of household wealth gap on individuals’ mental health with different age, education and household registration backgrounds, which is conducive to understanding the heterogeneity of the impact of household wealth gap on individuals’ mental health. Third, this study found that individuals’ health investment and neighborhood relationships are the mechanisms by which family wealth gap affect individual mental health, which provides policy implications for governments and households to improve individuals’ mental health.

The remainder of this study is organized as follows: Chap. 2 presents the theoretical analysis and research hypotheses. Chapter 3 explains the data sources and econometric model. Chapter 4 reports the results of the empirical analysis. Chapter 5 discusses the results of this study. Chapter 6 presents the conclusion.

## Theoretical analysis and research hypothesis

In this section, we introduce social comparison and relative deprivation theories. Furthermore, we analyze the impact of household wealth gap on individuals’ mental health based on these two theories.

Festinger (1954) first proposed the social comparison theory [13]. The basic idea of this theory is that humans have social attributes, and social comparison is individuals’ spontaneous and unconscious behavior. Everyone wants to know, consciously or unconsciously, how capable one is and what one’s status is. Only by comparing themselves with others in society can one truly know themselves and find the gap between them and others. Relative deprivation was first proposed by the American scholar Stouffer, and Merton further extended it to become a theory of group behavior. Relative deprivation is the feeling of deprivation that occurs when an individual compares one’s situation with a reference or a standard and finds themselves at a disadvantage. Relative deprivation is a feeling everyone is entitled to but does not possess.

The impact of household wealth gap on individual’s health can be partially explained by social comparison theory. From social comparison theory, it is known that people will consciously or unconsciously compare themselves with the groups around them. Individuals make comparisons in two ways: horizontal comparisons and vertical comparisons. The horizontal comparison emphasizes comparing oneself with other individuals in the frame of reference based on one’s conditions and rewards. Vertical comparison means individuals compares one’s current efforts and rewards with one’s past. Humans are born to be fair, and unfair encounters can cause negative emotions. The impact of wealth on individual’s health depends on the results of comparing one’s wealth with that of others. Individuals with wealth at the top of the pyramid are paid far more than the average resident simply by inheritance, capital reinvestment, or property income, leading to a greater sense of inequity felt by other individuals compared to them in a reference group. This inequity further leads to negative emotions such as frustration and anger, which may affect their mental health.

Relative deprivation theory can also explain the negative impact of household wealth gap on individual’s health to a certain degree. Relative deprivation theory suggests that people feel unequal and exploited when they find themselves in a disadvantageous position compared to others in a reference group. Thus, the choice of reference group determines whether an individual feels deprived. It is generally believed that people tend to compare upward, meaning that individuals will compare themselves with those in the reference group who have higher wealth than they do. Therefore, the larger the wealth gap, the stronger the relative deprivation felt by the individual will be. This deprivation will lead to negative emotions such as dissatisfaction, pessimism, or anger, affecting the individual’s health status. In summary, this study proposes the following hypothesis.

### Hypothesis 1

Household wealth gap may negatively impact individuals’ mental health.

As an important health insurance, medicare enables enrollees to receive higher-quality health care [14]. At the same time, it has been shown that medicare can improve the individuals’ health by increasing their financial accessibility [15]. Patients with chronic diseases require long-term medical care and medication regimens. Medicare can reduce the medical burden of chronic disease patients and alleviate individuals’ concerns about medication access, thereby improving their health status. In addition, Lei and Lin (2009) indicate that participation in the new rural cooperative medical insurance significantly increased participants’ use of routine medical checkups and preventive medical services [16]. Sun and Lyu (2020) found that participation in the new rural cooperative medical insurance significantly positively affected adults’ self-rated health and mental health [17]. Meng et al. (2018) showed that medical insurance improved the individual’s health. Therefore, health insurance can improve the individual’s health [18].

Household wealth inequality may impact individual’s investment in health insurance. As the wealth gap increases, groups at the top of the wealth chain hold more concentrated resources and wealth. Taking advantage of that, they can further widen the wealth gap between them and the poorer individuals, thereby accumulating wealth and gaining higher status. This concentration of wealth leads to a higher percentage of the population being considered poor. Widening the wealth gap may lead to the Matthew effect of ‘the poor get poorer, and the rich get richer.‘ Residents at the tail end of the wealth distribution tend to face significant budget constraints when purchasing health insurance and even find basic livelihood security problematic.

Widening the household wealth gap may influence the individual’s health insurance investment through information channels. In areas with large wealth gap, wealth and power may be concentrated in the hands of a few individuals at the top of the wealth chain [19]. This group will take up more resources and reduce the rational allocation of healthcare resources. As the gap in household wealth widen, it will lead to a divergence in social capital and access to information between the poor and the rich. The wealthy have higher social capital and can quickly and accurately obtain information about health investments. The information advantage and social capital give the rich better access to healthcare resources. Due to information asymmetry, it is difficult for the poor obtain various resources, such as healthcare and education.

In addition, it has also been found that the widening of the household wealth gap affects individuals’ insurance investments by harming their trust in government or public health institutions. For example, previous study showed that increased inequality might lead to political inequity in participation and devalue people’s trust in government or public institutions, reducing the willingness of society members to participate in health insurance [20]. In summary, a wider wealth gap may reduce the willingness of residents to purchase health insurance. Based on the above analysis, this study proposes the following hypothesis.

### Hypothesis 2

The household wealth gap may affect individuals’ mental health by reducing their health insurance investment.

Neighborhood enhancement may have a significant positive impact on individuals’ health through different pathways. Among the community social environment factors, good neighborhood relations may manifest as fewer neighborhood disputes, more diverse community organizational groups, more frequent and close neighborhood interactions, and a good environment for neighborhood interactions, significantly contributing to individuals’ health levels. Previous study showed that neighborhood relations might improve individuals’ health by influencing health information dissemination, increasing individuals’ participation in social groups or group activities (e.g., physical exercise), and control of unhealthy social behaviors improving individuals’ health status [21]. Diffusion of innovation theory suggests that innovative behaviors (e.g., use of preventive services) spread more quickly in cohesive communities whose members know and trust each other. Thus, information about healthy living is better disseminated in social networks with close neighborhoods, improving individuals’ health.

Neighborhoods may also influence individuals’ health by increasing their social engagement. Previous studies have found that community environments with more friendly neighborhoods significantly improve the health of residents. For example, Zeng et al. (2010) [22] showed that daily community activities and physical activity increased the physical fitness of residents in the community. In addition, communication and interaction with households, friends, and neighbors can alleviate individuals’ inner loneliness, positively impacting their health. In fact, for individuals who do not have access to spousal care and mental comfort, enhancing neighborhood relationships is even more important [23]. Regular interaction with neighbors and exercise will greatly improve the health status of these unaccompanied individuals. Good neighborly relationships can also provide daily care for the elderly, who lack the companionship and care of their children. They can effectively relieve their physical and mental fatigue and gain mental comfort by visiting their neighbors, thus improving their health. In conclusion, good and harmonious neighborhood relations can improve individuals’ health through information exchange, daily care, relief of loneliness, and physical exercise.

The household wealth gap can harm neighborhood relations. Specifically, the widening gap in household wealth can lead to negative feelings of wealth hatred, jealousy, and relative deprivation among poor individuals, leading to more distant relationships between neighbors. First, for those families at the end of the wealth spectrum, the widening wealth gap may stimulate “wealth hatred” among families. Those families at the middle and upper wealth levels may look down on those at the bottom who lack wealth accumulation, which may lead to conflicts. In addition, some individuals often compare themselves with each other in their daily interactions with their neighbors. They tend to feel disadvantaged if they are financially stressed, and this negative emotion will produce extremely serious harm [24]. These negative emotions may affect individuals’ work and other aspects of their lives. It may also reduce their tolerance and patience, leading to bad behaviors such as temper tantrums and anger, thus affecting interpersonal interactions. At the same time, increased inequality can also lead to a sense of unfairness. This psychology may lead to great anxiety, depression, and other adverse psychological stress emotions, affecting neighborhood relations.

The widening gap in household wealth also manifests in the differentiation of social classes. Previous studies have shown that residents of the upper and middle social classes are more likely to be able to acquire wealth through legitimate institutional measures than residents of the lower social classes, which can lead to a sense of relative deprivation of social wealth sprouting in households at the bottom of the social ladder. People with a higher sense of relative deprivation are less inclined to help others, have a lower willingness to get closer to society, are more isolated in their daily social life, and do not spend time and energy on maintaining good neighborhood relations [25]. At the same time, they are prone to non-safe and addictive behaviors such as gambling, alcoholism, smoking, and worse health [26, 27], which in severe cases can even lead to malignant group events such as crime and rebellion. Out of concern for their safety and self-protection, wealthy families in the upper social class will reduce or even refuse to interact with their neighbors in the lower social class, which will inevitably create a gap and affect neighborhood solidarity. Therefore, the widening gap in household wealth may worsen neighborhood relations. Based on the above analysis, this study proposes the following hypothesis.

### Hypothesis 3

Increasing the household wealth gap may affect individuals’ mental health by affecting neighborhood relations.

## Data source and methodology

### Data

The data used in this study are mainly from the China Family Panel Survey (CFPS) implemented by the China Social Science Survey Center (ISSS) of Peking University. The CFPS sample is very representative, covering 25 developed and less developed provinces/autonomous regions, such as Sichuan, Zhejiang, and Guangdong, with a large sample size, wide coverage, and scientific sampling methods. Meanwhile, to better control the estimation bias caused by omitted variables, this study also controls macro variables such as healthcare fiscal expenditure, economic development level, and inclusive financial development. Among them, the macro data of health care fiscal expenditure and economic development level are obtained from the Wind database. The level of financial inclusion development is measured using the Peking University Digital Financial Inclusion Index. In terms of data processing, samples with outliers were removed from this study to prevent the influence of outliers on the estimation results. Since the reference frame in calculating the household wealth gap is the village/household, the samples with missing individual village/household information are removed from this study. At the same time, considering that the measure of the household wealth gap needs to have a frame of reference, and the household wealth gap in this study is at the village/community level, the sample with only one interviewed individual at the village/community level is deleted in this study. Finally, the samples with missing variables are also removed in this study.

### Variable definition

#### Dependent variable

The explanatory variable in this study is mental health. Referring to the study of Zhang et al. (2022) [28], this study based on the 8-question CES-D scale in the CFPS to measure individuals’ mental health^1^. The 8 questions asked in the CFPS are the frequency of the following feelings or behaviors over the past week (1) I feel depressed; (2) I find it hard to do anything; (3) I do not sleep well; (4) I feel happy; (5) I feel lonely; (6) I live a happy life; (7) I feel sad and upset; (8) I feel that I cannot continue my life. Respondents could answer (a) hardly ever (less than one day); (b) some of the time (1–2 days); (c) often (3–4 days); (d) most of the time (5–7 days). For the six questions (1), (2), (3), (5), (7), and (8), the responses of a, b, c, and d were assigned as 4, 3, 2, and 1, respectively. For questions (4) and (6), the responses of a, b, c, and d were assigned as 1, 2, 3, and 4, respectively. The above treatment ensures that the higher the respondent’s score, the better individual’s mental health status. By summing up the scores of the 8 questions, we can obtain the level of individuals’ mental health, and the higher the value, the better the mental health of the individual.

#### Explanatory variable

This study uses the Kakwani index to measure the wealth gap of households^2^. The Kakwani index is not only a micro-indicator of the degree of inequality but also an alternative measure of relative wealth. The Kakwani index is widely used in studies related to income distribution [9, 24, 30–32]. The Kakwani index corresponding to the individual’s household *k* is

$$RW(y,{y_k})=\frac{1}{{{N_i}\mu }}\sum\limits_{{i=k+1}}^{{{N_i}}} {({y_i}} - {y_k})$$

The variable ${y_k}$ represents the wealth of the household *k*; the variable ${y_i}$ represents the wealth of a richer household *i* in the reference group. There are ${N_i}$ other households with higher wealth than the individual’s household, who form the reference group for the household *k*. The variable $\mu$ represents the mean value of the wealth of the households in the reference group. By taking the average of differences between the wealth of the household *k* and each household from the reference group, the Kakwani index is obtained. This index reflects the inequality of wealth among households. The larger the index, the greater the wealth gap between the individual’s household and its reference group.

#### Control variable

To examine the impact of the household wealth gap on individual’s mental health, this study further controls for the corresponding individual characteristics, household characteristics, and some characteristics of the province where the individual is located to ensure the reliability of the estimated results. Specifically, those characteristics include the individual’s age, education level, marital status, household registration, annual income, whether the individual smokes, household size, household expenditure, whether family member is involved in agricultural production, whether family member is engaged in entrepreneurship, whether the family receives government subsidies, whether the family has experienced demolition, finance expenditure on health care, economic development, and the development of financial inclusion. The definitions of each variable are presented in Table 1.

**Table 1:** Variable definitions

Variable Type	Variable Name	Variable Definition
Explained variable	Mental Health	Calculated based on the 8-question CES-D scale
Explanatory variable	Household wealth gap	Calculated by the Kakwani index
Control variables	Age	The age of individual
	Education level	The education year of individual
	Marital Status	Married is assigned the value of 1, and 0 otherwise
	Household Registration	Urban household registration is assigned a value of 1, and 0 otherwise
	Income	The total annual income of the individual (in logarithm)
	Smoking	Smoking is assigned a value of 1, and 0 otherwise
	Household size	Number of members in the household
	Household expenditure	Annual household consumption expenditure (in logarithm)
	Agriculture	If a family member is involved in agricultural production, the value is assigned as 1, and 0 otherwise.
	Entrepreneurship	If a family member is engaged in entrepreneurship, assign a value of 1, and 0 otherwise.
	Subsidy	If the family receives government subsidies, assign a value of 1, and 0 otherwise.
	Demolition	If the family has experienced demolition, assign a value of 1, and 0 otherwise.
	Finance expenditures	Provincial-level health care finance expenditure (in logarithm)
	Economic development	Per capita GDP of provinces (in logarithm)
	Inclusive Finance	Province-level financial inclusion index (in logarithm)

#### Descriptive statistical analysis of variables

Table 2 reports descriptive statistical analysis results for the mental health and household wealth gap. Given the large values of individuals’ income, health care financial expenditure, economic development level, and financial inclusion index, these variables take the logarithmic transform in this study. The descriptive statistical analysis results in Table 2 show that the effective sample size of the mental health variable is 63,524, with a mean value of 26.979, a minimum value of 8, and a maximum value of 32. The sample size for the household wealth gap variable is larger since extra data from 2014 are collected. The maximum and minimum values of the variables are within reasonable limits, and there are no significant outliers.

**Table 2:** Descriptive statistical analysis of variables

Variable	Observation		Mean	Standard deviation	Minimum	Maximum
Mental health	63,524	26.979		3.918	8.000	32.000
Household wealth gap	85,638	0.593		2.747	0.000	601.000
Age	85,638	53.270		19.184	16.000	120.000
Education level	85,638	6.983		4.917	0.000	22.000
Marital status	85,638	0.830		0.376	0.000	1.000
Household registration	85,638	0.454		0.498	0.000	1.000
Income	85,638	3.046		4.595	0.000	16.148
Smoking	85,638	0.294		0.455	0.000	1.000
Household size	85,638	4.355		1.989	1.000	21.000
Household expenditure	85,638	10.666		0.911	2.303	15.458
Agriculture	85,638	0.581		0.493	0.000	1.000
Entrepreneurship	85,638	0.109		0.312	0.000	1.000
Subsidy	85,638	0.522		0.500	0.000	1.000
Demolition	85,638	0.013		0.115	0.000	1.000
Finance expenditure	85,638	24.419		0.531	22.599	25.670
Economic development	85,638	10.732		0.414	9.889	11.851
Inclusive finance	85,638	5.240		0.415	4.329	5.934

### Methodology

This study first examines the impact of the household wealth gap on individuals’ mental health using the two-way fixed effect (TWFE) model, which is set up as follows:

1 $$Healt{h_{it}}=\rho +{\beta _1}Wealthga{p_{it}}+\sum\limits_{{}}^{{}} {Contro{l_{it}}} +{\mu _i}+{\lambda _t}+{\varepsilon _{it}}$$

Variable $Healt{h_{it}}$ is the dependent explanatory variable: individuals’ mental health. $\rho$ is the constant term for model estimation. Variable $Wealthga{p_{it}}$ is the core explanatory variable representing the household wealth gap. Coefficient ${\beta _1}$ is the estimation coefficient of the impact of the household wealth gap on individuals’ mental health. $Contro{l_{it}}$ is a set of control variables that may affect both the individual’s mental health and the household wealth gap. Parameter ${\mu _i}$ is the error terms that do not vary over time. Parameter ${\lambda _t}$ represents year-fixed effect, and parameter${\varepsilon _{it}}$ represents random perturbation term that vary over individuals and time.

## Empirical results

### Baseline regression results

Table 3 reports the impact of household wealth gap on individual’s mental health. The estimation results in columns (1) and (2) of Table 3 show that the estimated coefficients of the household wealth gap are negatively significant whether or not include variables such as age, education level, and other household-level and province-level variable. It indicates that the household wealth gap significantly negatively affects individual’s mental health. Analysis of the estimated coefficients of the control variables shows that marital status, household size, and economic development significantly positively affect the individual’s mental health. Smoking has a significant negative impact on an individual’s mental health.

**Table 3:** Impact of household wealth gap on individuals’ mental health

Variable	(1)	(2)
	Mental Health	Mental Health
Household wealth gap	-0.2081**	-0.1935**
	(0.0904)	(0.0904)
Age		-0.0049
		(0.0064)
Education level		0.0146
		(0.0292)
Marital status		1.0150***
		(0.1583)
Household registration		-0.0227
		(0.1585)
Income		0.0026
		(0.0060)
Smoking		-0.2083*
		(0.1150)
Household size		0.0503*
		(0.0261)
Household expenditure		-0.0526
		(0.0408)
Agriculture		0.0589
		(0.0917)
Entrepreneurship		0.0596
		(0.1053)
Subsidy		0.0561
		(0.0701)
Demolition		0.0833
		(0.2005)
Finance expenditure		0.2723
		(0.5216)
Economic development		0.9208**
		(0.3835)
Inclusive finance		0.1031
		(0.4143)
Individual fixed effects	YES	YES
Year fixed effect	YES	YES
Constant	27.0909***	9.6269
	(0.0506)	(10.8570)
Sample size	63,524	63,524
R-squared	0.0375	0.0425

Note: * p < 0.10, ** p < 0.05, *** p < 0.01. The standard error (clustering to household level) is reported in parentheses

### Consider endogeneity

There may be endogeneity issues in estimating the impact of the household wealth gap on individuals’ mental health. First, it is impossible to control all variables affecting the household wealth gap and health. In addition, individuals may underestimate or overestimate their mental health status, leading to measurement errors.

Using the two-stage least square (2SLS) to examine the impact of household wealth inequality on individuals’ mental health, we need to find the instrumental variable (IV) of the household wealth gap. This study constructs an interaction term based on the house price at the province level in the individuals’ region and the inequality index for the number of houses in that province. This study uses this interaction term as the IV for the household wealth gap. The construction of the inequality index for the number of houses is based on the number of houses owned by each household in each survey year in the CFPS from 2012 to 2018. Moreover, the specific measure is obtained by referring to the formula of the Gini coefficient. By calculating the average price of housing in cities under the jurisdiction of different provinces in different years, the average price of housing in the province where the individual lives can be obtained.

There are several reasons for constructing IV based on house prices and the inequality index for the number of houses. First, previous studies have found that high house prices can increase the asset value of the middle class but may make it more difficult for poorer groups to obtain their first house. Thus, it can widen the wealth gap between the poor and middle classes [33]. Although some studies have suggested that house prices may reduce the household wealth gap [8], this is the opposite case in China. Previous studies show that property value contributes as high as 70% to the wealth gap in China, and the rate is gradually increasing [2, 34]. At the same time, the above studies suggest that the rise in house prices has driven the wealthy class to invest more in property assets, further widening the wealth inequality. On the provincial level, a higher inequality index for the number of houses leads to a wider household wealth gap through property wealth inequality. The degree of wealth inequality can be well explained by multiplying house prices with the inequality index for the number of households.

Second, a perfect instrumental variable needs to satisfy the requirement of exogeneity. That is, the instrumental variable can only indirectly affect individuals’ health by influencing the household wealth gap, but not through other aspects. The above interaction term constructed in this study does not directly affect individuals’ mental health, which satisfies the condition of an instrumental variable. When there are more than two instrumental variables, the “overidentification test” can be used to verify the exogeneity of the instrumental variables. However, when the number of instrumental and endogenous variables are equal, the exogeneity of the instrumental variables cannot be tested by statistical methods. Therefore, this study regresses both the constructed instrumental variable and the household wealth gap on the individual’s mental health. Suppose the instrumental variable only indirectly affects individuals’ mental health by affecting the household wealth gap. In that case, the effect of the instrumental variable on individuals’ mental health should be insignificant when controlling for the household wealth gap. The estimation results in column (1) of Table 4 show that the effect of instrumental variable on individuals’ mental health is insignificant after controlling for the household wealth gap, indicating that the instrumental variable satisfies exclusivity.

This study also uses statistical tests to examine the correlation between instrumental variable and endogenous variables. The Wald test with a “nominal significance level” of 5% was conducted for the weak instrumental variables. The first stage of the 2SLS estimation result was used to determine whether there was a weak instrumental variable problem. The “minimum characteristic statistics” of the Wald test are greater than the critical value of 8.96, and the F-value of the first stage of 2SLS was 194.13, much higher than the critical value of 10. The 2SLS first-stage estimation results in column (2) of Table 4 indicate that the estimated coefficients of the effects of the instrumental variable on the household wealth gap are positively significant. All the above results indicate that there is no weak instrumental variable problem. In summary, using the interaction term as the instrumental variable is appropriate.

The estimation results of 2SLS in column (3) of Table 4 show that the estimated coefficient of the household wealth gap is -1.2483 after accounting for endogeneity. The coefficient is negatively significant at the 1% significance level, indicating that the household wealth gap significantly negatively impact individuals’ mental health. Considering that the endogeneity issue may cause estimation bias, the baseline results are estimated using column (3) of Table 4 when analyzing the robustness, heterogeneity, and mechanism of the impact of household wealth gap on individuals’ health in the later study.

**Table 4:** Impact of the household wealth gap on individual’s mental health (considering endogeneity)

Variable	(1)	(2)	(3)
	Exclusivity test	Phase I	Phase II
	Mental Health	Wealth Gap	Mental Health
Household wealth gap	-0.6403***		-1.2483***
	(0.0448)		(0.2395)
Instrumental variable	-0.0094	0.0345***	
	(0.0076)	(0.0010)	
Control variable	YES	YES	YES
Year fixed effect	YES	YES	YES
Constant	36.1492***	1.8133***	10.3763***
	(7.5783)	(0.1478)	(1.3649)
Sample size	63,524	63,524	63,524

Note: *** p < 0.01. Standard errors are clustered at the household level in parentheses

### Robustness check

#### Robustness test I: replacing the instrumental variable

In the above study, we constructed an interaction term as an instrumental variable of the household wealth gap based on the house price and housing inequality index. To ensure the robustness of the estimation results, this study refers to previous research, uses the one-period lag of the wealth gap as an instrumental variable, and uses 2SLS to examine the impact of the household wealth gap on individual’s mental health^3^. The results in column (1) of Table 5 show that the estimated coefficient of the impact of household wealth gap on individuals’ mental health remains negatively significant, which indicate that household wealth gap can have a significant negative impact on individual’s mental health.

**Table 5:** Estimation results of robustness check

Variable	(1)	(2)	(3)	(4)
	Mental Health	Mental Health	Mental Health	Mental Health
Wealth gap	-0.3323***	-0.3842***	-0.2030***	-0.2639***
	(0.0369)	(0.0715)	(0.0419)	(0.0497)
Control variable	YES	YES	YES	YES
Year fixed effect	YES	YES	YES	YES
Constant	-2.0559***	-4.4208***	-2.9110***	-3.1872***
	(0.6229)	(0.3408)	(0.2352)	(0.2817)
Sample size	30,120	63,524	63,524	63,524

Note: *** p < 0.01. Standard errors are clustered at the household level in parentheses

#### Robustness test II: changing the measurement of the explanatory variable

In this study, a dummy variable is constructed to characterize individuals’ mental health. If the individuals’ mental health score is greater than the mean of the mental health level scores of the entire sample, the dummy variable is assigned a value of 1 and 0 otherwise. Given that the dependent variable is a dummy variable, the IV-Probit model is used to examine the impact of household wealth gap on the individual’s mental health. From the estimated results of the IV-Probit model in column (2) of Table 5, it is clear that the household wealth gap significantly negatively affects individuals’ mental health.

#### Robustness test III: changing the measurement of core explanatory variables

To alleviate the possible estimation bias caused by direct summation, referring to Zhang et al. (2022) [28], factor analysis and principal component analysis were also used to measure the individual’s mental health. The applicability of factor analysis and the principal-component analysis was also tested in this study. The KMO values of each variable were greater than 0.8 for both principal component and factor analysis. Also, the results of Bartlett’s spherical test rejected the original hypothesis of no correlation between variables (Cronbach alpha greater than 0.7). These results indicate that principal-component and factor analysis is appropriate for measuring individuals’ psychological well-being. The higher the score, the better the mental health of the individual. Based on the above two measures of the individual’s mental health, this study uses 2SLS to examine the impact of household wealth gap on individual’s mental health. From the estimated results in columns (3) and (4) of Table 5, we can see that the estimated coefficients of the household wealth gap are both negatively significant, further indicating the robustness of our conclusion.

#### Robustness test IV: replacement of the measure of the wealth gap

There are different approaches to measure the household wealth gap in the literature. This study uses the 90th percentile household net wealth value minus the 10th percentile household net wealth value for each village as a proxy variable for the household wealth gap. In addition, this study uses the value of net household wealth at the 75th percentile minus the value of net household wealth at the 25th percentile for each village as a proxy for the household wealth gap. This study uses 2SLS to examine the impact of the household wealth gap on individual’s mental health. As shown by the estimated results in columns (1) and (2) of Table 6, the estimated coefficients of household wealth gap are both negatively significant, further indicating the robustness of our conclusion.

In addition, this study also uses the Yitzhaki index to measure the household wealth gap. The Yitzhaki index differs from the Kakwani index in that the Yitzhaki index does not consider the mean wealth of the reference group. Column (3) of Table 6 results show that the estimated coefficient of the impact remains negatively significant, further indicating the robustness of our findings.

**Table 6:** Robustness tests: replacing the measurement of household wealth gap

Variable	(1)	(2)	(3)
	Mental Health	Mental Health	Mental Health
P90-P10	-0.0182***		
	(0.0055)		
P75-P25		-0.0521***	
		(0.0199)	
Yitzhaki_index			-0.0281***
			(0.0085)
Control variable	YES	YES	YES
Year fixed effect	YES	YES	YES
Constant	-15.0835**	-16.9789*	-9.4789
	(7.1473)	(9.8063)	(5.8206)
Sample size	63,524	63,524	63,524

Note: * p < 0.05;** p < 0.05;*** p < 0.01. Standard errors are clustered at the household level in parentheses

#### Robustness test V: Considering the self-selection problem

Individuals may choose their community based on their health status and thus have a self-selection problem. Considering that self-selection may make the estimation results biased and create endogeneity problems. Therefore, this study examines the impact of the household wealth gap on individuals’ mental health using propensity score matching, referring to the study by Zhang et al. (2022) [28]. Specifically, this study constructs a dummy variable for the level of the wealth gap, which is assigned a value of 1 if the household wealth gap is greater than the mean of all household wealth gap levels and 0 otherwise. The average treatment effect (ATT) can be calculated using propensity score matching. If the average treatment effect is negatively significant, it indicates that the household wealth gap significantly negatively affects individuals’ mental health.

**Fig. 1:**
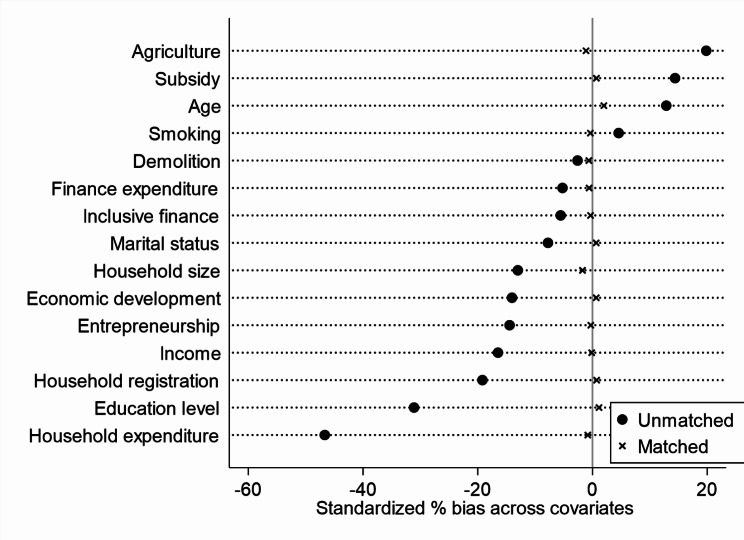
Balance test

**Fig. 2:**
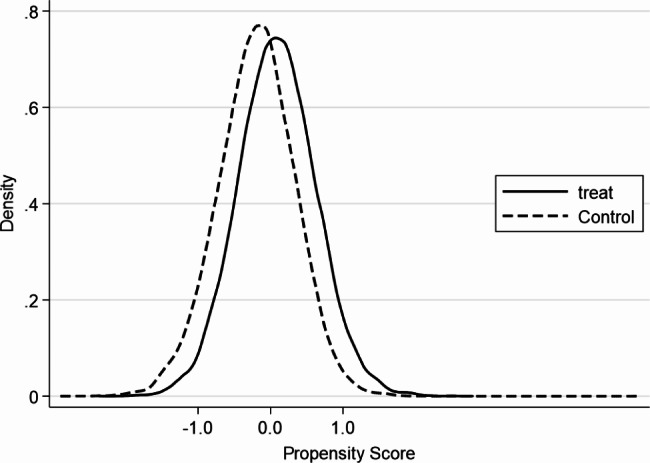
Fitting plots of propensity scores for the treatment and control groups before matching

**Fig. 3:**
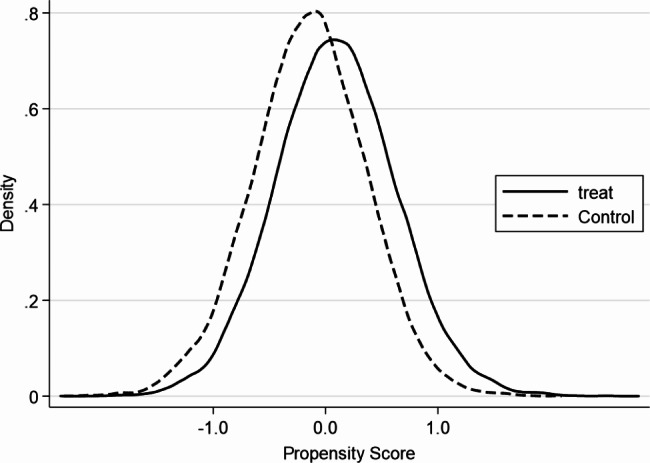
Fitting plots of propensity scores for the treatment and control groups after matching

Before using PSM to estimate the impact of household wealth gap on individuals’ mental health, this study conducted a balance test. As can be seen from Fig. 1, the standardized deviations after matching were all less than 10%, indicating that the matching passed the balance test (Fig. 1 reports the distribution of standardized deviations for each variable obtained using K-nearest neighbor matching (K = 4)). Figures 2 and 3 show that the probability density plots of propensity scores for the treatment and control groups before and after matching have a large overlapping portion, indicating that the common support assumption is satisfied. The above results suggest that it is appropriate to use PSM to investigate the impact of the household wealth gap on individuals’ mental health.

To ensure the robustness of the estimation results, referring to Zhang et al. (2022) [28], different matching methods such as K-nearest neighbor (K = 1, K = 4), local linear regression matching, radius matching, and kernel matching were used to examine the effect of household wealth gap on individuals’ mental health. As shown by the estimation results in Table 7, the ATT of any matching methods is negatively significant, indicating that the household wealth gap has a significant negative impact on individuals’ psychological well-being after considering self-selection, further illustrating the robustness of the estimation results above^4^ .

**Table 7:** Effect of household wealth gap on individuals’ health (considering self-selection)

Variable	(1)	(2)	(3)	(4)	(5)
	K = 1 nearest neighbor	K = 4 near neighbor	Local linear regression matching	Radius Matching	Kernal matching
ATT	-0.382***	-0.380***	-0.3874 ***	-0.4055***	-0.4346***
	(0.044)	(0.0362)	(0.0435)	(0.0336)	(0.033)
Sample size	63,524	63,524	63,524	63,524	63,524

Note: * p < 0.10, ** p < 0.05, *** p < 0.01. Standard errors of coefficients are reported in parentheses

### Heterogeneity analysis

This study further examines the heterogeneity of the impact of household wealth gap on individual’s mental health with different levels of education, household registration and age group, respectively.

#### Heterogeneity in educational attainment

This study divided the sample into two subsamples with high and low levels of education, and examined the impact of the household wealth gap on the mental health of individuals with the two levels of education using two-stage least squares (2SLS), respectively^5^. From the estimated results in Table 8, it can be seen that the estimated coefficients of household wealth gap in highly and poorly educated group are negatively significant, indicating that the household wealth gap has a significant negative impact on the health of individuals with different levels of education. Analysis shows that the absolute values of the estimated coefficients of the impact with lower education levels are greater than those with higher education levels. In conclusion, the impact of household wealth gap on the health of individuals with low education levels is greater than that of individuals with high education levels.

**Table 8:** Heterogeneity analysis results

Variable	(1)	(2)	(3)	(4)	(5)	(6)
	Mental Health		Mental Health		Mental Health	
	High education level	Low education level	Urban	Rural	Age < 45	Age > 44
Wealth gap	-1.090***	-1.975***	-0.740**	-1.811***	-1.142***	-1.245***
	(0.260)	(0.417)	(0.292)	(0.396)	(0.402)	(0.275)
Control variable	YES	YES	YES	YES	YES	YES
Year fixed effect	YES	YES	YES	YES	YES	YES
Constant	17.651***	5.793***	10.615***	11.926***	17.869***	7.287***
	(1.586)	(2.114)	(1.861)	(2.074)	(2.611)	(1.570)
Sample size	32,213	31,311	28,596	34,928	18,128	45,396

Note: *** p < 0.01. Standard errors are clustered at the household level in parentheses

#### Heterogeneity of household registration

This study examines the heterogeneity of residents with different household registration. The estimation results in columns (3) and (4) of Table 8 show that the impact of household wealth gap on the mental health of residents in both urban and rural areas is negatively significant. The absolute value of the estimated coefficient of the impact on the mental health of residents in urban areas is smaller than that in rural areas. These results indicate that the impact of household wealth gap on rural residents’ mental health is greater than that of urban areas.

#### Heterogeneity of age

Considering that the impact of family wealth gap on the mental health of individuals in different birth cohorts may vary, this study further investigates the influence of family wealth gap on the mental health of individuals across different age groups. Specifically, this study divides the sample into two groups: middle-aged and elderly (Age 45 and above), and youth (age below 45), based on age. This study employs a 2SLS model to examine the effect of wealth inequality on the mental health of individuals across different age groups. The estimated results in columns (5) and (6) of Table 8 reveal a statistically significant negative impact of family wealth gap on the mental health in different age groups. The absolute value of the estimated coefficient for household wealth gap in column (3) is smaller than the absolute value of the estimated coefficient for household wealth in column (4). These findings suggest that the impact of family wealth gap on the mental health of middle-aged and elderly individuals is greater compared to young individuals.

### Mechanism analysis

#### Impact of household wealth gap on individual’s health insurance investment

In the theoretical analysis, we have analyzed how the household wealth gap may affect individuals’ health by influencing health insurance investment. Here, we examine the impact of the household wealth gap on health insurance investment by using whether individuals purchase health insurance as a proxy variable for health insurance investment and then verify the mechanism by which the household wealth gap affects health from an empirical perspective. Column (1) of Table 9 reports the impact of the household wealth gap on the individual’s health insurance investment (assigned a value of 1 if they have purchased health insurance and 0 otherwise)^6^.

The estimation results in column (1) of Table 9 indicate a significant negative impact of the household wealth gap on the purchase of health insurance, meaning that the household wealth gap can significantly reduce the likelihood of purchasing health insurance. The column (2) of Table 9 presents the estimated effect of household wealth gap on individual mental health, taking into account health insurance investment in the benchmark regression model. The results show that the absolute value of the coefficient for wealth gap has slightly decreased compared to Table 3. Additionally, the estimated coefficient for the impact of health insurance investment on individual mental health is positively significant. The above findings are consistent with the results obtained from the theoretical analysis above, further suggesting that the household wealth gap can affect individuals’ mental health by reducing individuals’ health insurance investment.

#### Impact of household wealth gap on neighborhood relations

Section 2 clarifies the mechanisms by which the household wealth gap may affect individuals’ health by influencing neighborhood relations at the theoretical level. To test hypothesis 3, firstly, we examine the influence of household wealth gap on neighborhood relationships. Secondly, we incorporate neighborhood relationships into the benchmark regression model to investigate the impact of wealth inequality on individual mental health. This study did not use the 2012 and 2018 CFPS data since neither contained relevant neighborhood information. The 2014 and 2016 CFPS data contain information about neighborhood relation, while they differ from each other. The specific question about neighborhoods from the 2014 question about neighborhood relationships is: “In the past 12 months, how was the relationship between your household and your neighbors?“ Respondents could answer “1. very amicable,“ “2. relatively amicable,“ “3. average (e.g., few interactions but no conflicts),“ “4. somewhat tense relationship,“ or “5. very tense relationship.“ The specific question about neighborhoods from the 2016 question about neighborhood relationships is: “How do you think the neighborhood relationship in your neighborhood is?“

Given that the 2014 CFPS question about neighborhood relations better reflects the relationship between individuals and neighborhoods. Hence, this study only uses the 2014 CFPS data. The estimation results in column (3) of Table 9 indicate that the household wealth gap significantly negatively impacts neighborhood relationship, suggesting that widening the household wealth gap can harm neighborhood relationship. In column (4) of Table 9, it is observed that the estimated coefficient for neighborhood relationship is found to be positively significant at the 1% level^7^. The above results are consistent with the theoretical analysis in Sect. 2, further suggesting that the household wealth gap can affect individuals’ mental health by influencing neighborhood relationship.

**Table 9:** Mechanism analysis results

Variable	(1)	(2)	(3)	(3)
	Health Insurance investment	Mental health	Neighborhood relationship	Mental health
Wealth gap	-0.0451***	-1.2007***	-0.0548**	-0.7724*
	(0.0158)	(0.2401)	(0.0266)	(0.4109)
Health insurance investment		0.2908***		
		(0.0611)		
Neighborhood relationship				0.4119***
				(0.0401)
Control variable	YES	YES	YES	YES
Year fixed effect	YES	YES	YES	YES
Constant	0.3823***	10.1348***	8.0717***	-9.4903***
	(0.0985)	(1.3654)	(0.5142)	(2.7062)
Sample size	85,436	63,353	22,113	22,113

Note: *** p < 0.01. Standard errors are clustered at the household level in parentheses

### Extended analysis

In this subsection, we further analyze the medium- and long-term impact of the household wealth gap on individual’s mental health. Given that CFPS data are surveyed every two years and that this study mainly uses CFPS data from 2012 to 2018 in this subsection, we introduce a lag of 2 years, 4 years, and 6 years for matching the individual’s health with the household wealth gap. Table 10 shows that the estimated coefficient of the impact of household wealth gap on the individual’s mental health after 6 years is -0.531, which is negatively significant at a 1% significance level. The impact of household wealth gap on the individual’s mental health after 2 years and after 4 years are both negatively significant. In summary, the household wealth gap not only affects the individual’s mental health in the short term but also has a significant negative impact on individual’s mental health in the medium and long term.

**Table 10:** Medium- and long-term effects of household wealth gap on individual’s health

Variable	(2)	(4)	(6)
	Mental Health	Mental Health	Mental Health
Wealth lag of 6 years	-0.531***		
	(0.123)		
Wealth lag of 4 years		-0.619***	
		(0.089)	
Wealth lag of 2 years			-0.732***
			(0.079)
Control variable	YES	YES	YES
Year fixed effect	NO	YES	YES
Constant	19.543***	8.726**	13.929***
	(4.512)	(3.604)	(3.404)
Sample size	10,359	24,786	30,120
R-squared	0.072	0.080	0.080

Note: *** p < 0.01. Standard errors are clustered at the household level in parentheses

## Discussion

Mental health has been a significant focal point in prior research endeavors. Previous studies have examined the determinants of individual mental health from different perspectives such as individual characteristics, environment, institutions, and government governance levels [35–39]. Additionally, previous research has delved into the effects of economic variables, such as income, income inequality, family wealth, and unemployment, on individual mental well-being [40–47]. Given that this study focuses on the impact of family wealth gap on individual’s mental health. Therefore, the most relevant literature to this study is the impact of inequality on individual health. Previous studies have examined the impact of income inequality and wealth inequality on individual health. In contrast to income, collecting data on household wealth presents greater challenges. Consequently, prior investigations have predominantly focused on examining the impact of income inequality on individual well-being. A substantial body of research has consistently demonstrated that income inequality can exert a noteworthy adverse influence on individual health [46, 48–50]. As the wealth gap widens across numerous countries, a considerable volume of research has shifted its attention towards examining the effects of household wealth inequality on the health of residents.

Due to data limitations, some research studies have not demonstrated the relationship between wealth inequality and mental health based on data [51–53]. The findings from these studies often struggle to establish the causal relationship between wealth inequality and health outcomes. To more accurately gauge the actual influence of wealth inequality on health, some research endeavors utilize varied datasets and employ empirical methodologies for more in-depth analysis. Previous studies examined the impact of household inequality on individuals’ health from macro and micro perspectives. Dierckens et al. (2020) [54] examined the impact of wealth inequality on adolescent mental health at the country level by using panel data from 17 countries, calculating an index of wealth inequality for each country, and matching it to the mean of adolescent mental health at the country level. The study showed that wealth inequality significantly negatively affects adolescent mental health. Nowatzki (2012) [12] examined the impact of wealth inequality on health in 14 more economically developed countries. The study found that countries with higher Gini coefficients had lower life expectancy and higher infant mortality rates. That is, wealth inequality significantly negatively affects the health of the residents.

As macro data may suffer from the problem of aggregate bias, to better identify the causal relationship between the household wealth gap and individuals’ health, many studies have examined the impact from a micro perspective. For example, Omer et al. (2014) [55] showed that wealth inequality significantly negatively affects the population’s physical health. Hong et al. (2006) [56] examined the effect of household wealth inequality on children’s physical development and nutritional status using multivariate logit regression based on Bangladesh Demographic Health Survey data with a sample of 5977 children born at 0–59 months of age. The study found that household wealth inequality was strongly associated with stunting in childhood. He et al. (2018) [57] examined the impact of wealth inequality on women’s self-rated health based on data from Nepal. Jaeggi (2021) [19] examined the impact of relative household wealth and community-level wealth inequality on individuals’ self-rated health, blood pressure, BMI, and respiratory disease in high-income countries. Their conclusion was that wealth inequality significantly affected the probability of individuals having respiratory diseases, hypertension, and self-rated health. Alaba and Chola (2014) [58] examined the effect of wealth inequality on obesity based on data from the National Income Dynamics Survey in South Africa. They found that wealth inequality had a significant positive effect on the prevalence of obesity. In summary, many studies have examined the impact of household wealth inequality on individuals’ physical health. Unlike the studies mentioned above, this study examines the impact of the household wealth gap on individuals’ mental health.

Some studies have also examined the impact of the wealth gap on individuals’ mental health based on microdata [59, 60]. For example, Smith et al. (2019) [59] examined the effects of objective and subjective relative wealth on mental health using a sample of 1620 individuals in rural southwestern Uganda. The study found that groups with a lower objective and subjective relative wealth had lower mental health than those with higher levels of wealth. Gibson-Davis and Hill (2021) [60] found that wealth inequality has a significant negative impact on children’s well-being based on Survey of Consumer Finances data. The study of Siegel (2003) [53] concentrates on women, and its conclusion highlights that the increase of wealth inequality can lead to a decline in individual mental well-being. Summarizing the preceding literature concerning the influence of inequality on individual health, it becomes evident that some studies have not demonstrated the relationship between wealth inequality and mental health based on data. Limited research employing data has concentrated on the Chinese population, and a substantial portion of studies has solely assessed the short-term impact of household wealth inequality on individual health, disregarding medium and long-term consequences. Moreover, prior research frequently overlooks the underlying mechanisms through which household wealth inequality can impact mental health.

This study found that household wealth gap can significantly negatively impact individuals’ mental health in China. The findings of this study are consistent with previous studies [52–54, 59, 61]. However, some studies have not conducted empirical analyses to assess the impact of wealth inequality on individual mental health [52, 53]. Furthermore, certain studies utilize macro-level data to explore the influence of wealth inequality on individual mental health [54, 59]. Different from the above research, this study based on microdata and can more accurately identify the causal relationship between household wealth gap and mental health. Although Marshall et al. (2014) [61] used microdata to examine the effect of inequality on individuals’ mental health, it was conducted on the elderly in the United Kingdom. In addition, none of these studies examined the medium- to long-term impact of the household wealth gap on individuals’ mental health. Furthermore, they did not consider the endogeneity of the household wealth gap and the mechanism of the impact. This study constructs instrumental variables for the household wealth gap, which better mitigates the possible endogeneity problem. This study examines the short-term and medium- to long-term impact of household wealth inequality on individuals’ mental health. We also clarify how the household wealth gap affects individuals’ mental health by influencing health insurance investment and neighborhood relationship. Compared to previous studies, this study is more comprehensive and the results are more convincing.

The results of the heterogeneity analysis indicate that the impact of the household wealth gap on the mental health of individuals varies based on their education levels, regions, and ages, exhibiting a notable heterogeneity. Specifically, the household wealth gap has a greater impact on individuals’ mental health with lower levels of education. The reason is that individuals with higher educational attainment tend to have higher human capital, and their income levels and employment quality are higher relative to those with lower educational attainment. In addition, the more educated group has advantages in housing, social security, and educational resources for their children, potentially mitigating the negative effect of relative deprivation caused by the wealth gap to some extent. At the same time, the individuals with higher educational attainment have higher health awareness and knowledge, and they are more willing to invest in health care such as health insurance. The increase in health investment can effectively improve the level of healthcare services, and improve their health status.

This study also shows that the impact of household wealth gap on the mental health of rural household residents is greater. Compared with urban areas, various resources, such as medical conditions, are more limited in rural areas. These limited resources tend to be concentrated in the hands of households with higher wealth. In contrast, urban areas have far better medical facilities and wider medical coverage than rural areas. Therefore, the wealth gap may have a greater impact on the health of residents in rural areas than in urban areas. The communication between residents of the same community in urban areas is far less compared with rural areas. In many cases, residents living in urban areas may not even know their neighbors or rarely communicate with each other, let alone compare themselves with their neighbors. On the other hand, in rural areas, the communication between residents is more frequently. Most of the residents in the village will bring gifts to each other and gather together during holidays, celebrations, or just the off-farming season. In frequent communication, it is naturally easier to compare with people around them. Those residents with lower household wealth are more likely to feel relative deprivation, which will cause psychological imbalance and even lead to mental illness in serious case.

The heterogeneity analysis results also revealed that the impact of wealth gap on the mental health of middle-aged and elderly individuals is greater compared to young individuals. This difference in impact may be attributed to the following reasons: firstly, middle-aged and elderly individuals may be more psychologically vulnerable compared to young people. Secondly, middle-aged and elderly individuals may engage in more social comparisons with their peers, particularly after retirement, when their social circle predominantly comprises individuals of similar age. Upon realizing that their wealth significantly lags behind others, they may experience feelings of inferiority, shame, and increased stress. Moreover, middle-aged and elderly individuals face greater pressure related to elderly care and medical expenses. As the wealth gap increases, they may become more concerned about their financial situation and their ability to meet their future retirement needs.

The results of the mechanism analysis indicate that the wealth gap among households could impact residents’ mental health through its influence on their investment in health insurance. Those results are consistent with hypothesis 2. The reasons can be explained as follows: first, health insurance may improve individuals’ mental health thought different channel [17, 18]. Secondly, the widening wealth gap within families may diminish trust in government or public health institutions, consequently lowering residents’ inclination to invest in insurance. Furthermore, our study has revealed that neighborhood relationship serves as one of the mechanisms through which household wealth gap impact individual mental health. Those results are consistent with hypothesis 3. The establishment of this mechanism can be attributed to the following reasons: the increase in wealth inequality may trigger a range of negative emotions, such as relative deprivation and feelings of jealousy among poor individuals. These emotions can have negative impact on their neighborhood relationships. The deterioration of neighborhood relationships can have a negative impact on individual mental health [21]. When neighborhood relationships decline, individuals may experience feelings of isolation, lack of support, and increased stress, which can contribute to a decline in their mental well-being.

This study also has some limitations. First, due to data accessibility, it is impossible to include all control variables that may affect both the household wealth gap and individuals’ mental health in the model. Second, considering the endogeneity of the household wealth gap, this study constructs an instrumental variable of the household wealth gap based on the house price and the inequality index for the number of houses, and examines the effect of household wealth gap on individuals’ mental health using 2SLS. However, the instrumental variable may have an impact on individual mental health by influencing living costs or economic development. In other words, the instrumental variable is not entirely exogenous. Future research can find more exogenous instrumental variables to identify the impact of the household wealth gap on individuals’ mental health. In addition, future research can examine the effect of household wealth gap on individuals’ mental health based on some quasi-natural experiments which may cause negative or positive shock to the household wealth gap.

This study has the following policy implications. First, the results in mechanism analysis indicate that household wealth gap has negative impact on individuals’ mental health by discouraging their investment in insurance and damaging neighborhood relationship. Therefore, the government should encourage residents to purchase health insurance, and give appropriate cash subsidies to households at the tail end of the wealth distribution. Regardless of the wealth of their neighbors around them, individuals should build harmonious and cordial neighborhood relationships. Second, the household wealth gap has a greater impact on lower educational, rural residents or middle age and elderly individuals. It is difficult for rural residents and residents with lower educational backgrounds to achieve wealth accumulation in China. Therefore, the government should insist on helping those residents to accumulate wealth. At the same time, the government should improve the pattern of income and wealth distribution, increase transfer payments to low- and middle-income groups, continuously raise the income of low-income groups and expand the group of middle-income. Lastly, given China’s significant aging population, it is crucial for the government to take measures to narrow the wealth gap between families. This approach would be conducive to achieving active aging and promoting overall societal well-being.

## Conclusion

Based on CFPS data from 2012 to 2018, this study examined the impact of the household wealth gap on individuals’ mental health using two-way fixed effects model and two-stage least squares. The findings show that household wealth gap have a significant negative impact on individual’s mental health. This study conducts robustness tests by replacing instrumental variable, replacing the measurement of explanatory variables, and considering self-selection. Various robustness tests support that the household wealth gap have a significant negative effect on individual’s mental health. In addition, this study examines the heterogeneity impact of household wealth gap on mental health of residents with different levels of education, household registration and age. The estimated results revealed that the impact of family wealth gap on mental health is more pronounced among individuals with lower education levels, rural residents, as well as middle-aged and elderly individuals. Our study also examines the mechanism of household wealth gap on individuals’ mental health. The mechanism analysis results show that the household wealth gap could affect individual’s mental health by influencing their health insurance investment and neighborhood relationship. Finally, this study analyzes the medium- and long-term impact of the household wealth gap on individual’s mental health. The estimation results indicate that the household wealth gap have significant negative impact on individuals’ mental health in the medium- and long-term.

## Data Availability

The datasets used and/or analysed during the current study are available from the following link: http://www.isss.pku.edu.cn/cfps/.

## Footnotes

1 The CES-D was developed by Radloff (1991) [29]. Currently, the CES-D scale is widely used in large surveys such as NHANES (National Health and Nutrition Examination Survey) and RHS (Health and Retirement Study), and is one of the most widely used scales for measuring mental health.

2 The selection of a reference group is particularly critical when using micro-level inequality indicators to measure wealth gap and to examine the impact of wealth gap on individuals’ mental health. Since the geographic scope of people’s daily life in rural areas is mainly villages, residents perceive and compare the differences between household wealth and other residents mainly within the geographic scope of villages. In urban areas, residents also spend a relatively larger proportion of their time in community-wide activities. Therefore, this study measures the household wealth gap by using other members of the same village/residence as the reference group.

3 When using a one-period lag of the household wealth gap as an instrumental variable for the wealth gap, some of the sample is lost because the panel data we used is unbalanced.

4 The sample size reported in Table 7 are the sample size used for matching.

5 An individual was considered to be highly educated if his or her education level was greater than the average number of years of education in the whole sample, otherwise, the individual was considered to be less educated.

6 In the CFPS questionnaire, the question related to the purchase of medical insurance for residents is: “Which medical insurance do you have”, respondents can answer “don’t know”, “public medical insurance”, “urban employee medical insurance”, “urban resident medical insurance”, “supplementary medical insurance”, “new rural cooperative medical insurance”, “None of the above”. The sample of “don’t know” was deleted from this study.

7 Given the absence of 8-question CES-D scale in the CFPS dataset for 2014 and the availability of neighborhood relationship data solely for that year, this study employed a 6-question CES-D scale to assess individual mental health while estimating the influence of neighborhood relationship and wealth gap on mental well-being.

## References

[1] Meng X (2007). Wealth accumulation and distribution in urban China[J]. Econ Dev Cult Change.

[2] Li S, Wan H (2015). Evolution of wealth inequality in China[J]. China Economic Journal.

[3] Credit Suisse’s Global Wealth Report. https://www.credit-suisse.com/about-us/en/reports-research/global-wealth-report.html (2021). Accessed 9 July 2023.

[4] Piketty T, Yang L, Zucman G (2019). Capital accumulation, private property, and rising inequality in China, 1978–2015[J]. Am Econ Rev.

[5] Keister LA, Moller S (2000). Wealth inequality in the United States[J]. Ann Rev Sociol.

[6] Fagereng A, Guiso L, Malacrino D (2016). Heterogeneity in returns to wealth and the measurement of wealth inequality[J]. Am Econ Rev.

[7] Henley A (1998). Changes in the distribution of housing wealth in Great Britain, 1985-91[J]. Economica.

[8] Kuhn M, Schularick M, Steins UI (2020). Income and wealth inequality in America, 1949–2016[J]. J Polit Econ.

[9] Lhila A, Simon KI (2010). Relative deprivation and child health in the USA[J]. Soc Sci Med.

[10] Subramanyam M, Kawachi I, Berkman L, et al. Relative deprivation in income and self-rated health in the United States[J]. Volume 69. Social science & medicine; 2009. pp. 327–34. 3. 10.1016/j.socscimed.2009.06.008 19552992

[11] Adjaye-Gbewonyo K, Kawachi I. Use of the Yitzhaki Index as a test of relative deprivation for health outcomes: a review of recent literature[J]. Volume 75. Social science & medicine; 2012. pp. 129–37. 1. 10.1016/j.socscimed.2012.03.004 22521678

[12] Nowatzki NR (2012). Wealth inequality and health: a political economy perspective[J]. Int J Health Serv.

[13] Festinger L (1954). A theory of social comparison processes[J]. Hum Relat.

[14] Wagstaff A, Lindelow M, Gao J (2009). Extending health insurance to the rural population: an impact evaluation of China’s new cooperative medical scheme [ J]. J Health Econ.

[15] Sommers BD, Gawande AA, Baicker K (2017). Health insurance coverage and health-what the recent evidence tells us[J]. N Engl J Med.

[16] Lei X, Lin W (2009). The New Cooperative Medical Scheme in rural China: does more coverage mean more service and better health?. Health Econ.

[17] Sun J, Lyu S (2020). Does health insurance lead to improvement of health status among chinese rural adults? Evidence from the China Household Panel Studies[J]. Int J Health Serv.

[18] Meng Y, Han J, Qin S (2018). The impact of health insurance policy on the health of the senior floating population-evidence from China[J]. Int J Environ Res Public Health.

[19] Jaeggi AV, Blackwell AD, Von Rueden C (2021). Do wealth and inequality associate with health in a small-scale subsistence society?[J]. Elife.

[20] Brehm J, Rahn W. Individual-level evidence for the causes and consequences of social capital[J]. Am J Polit Sci, 1997: 999–1023.

[21] Kawachi I, Kennedy BP, Glass R (1999). Social capital and self-rated health: a contextual analysis[J]. Am J Public Health.

[22] Zeng Y, Gu D, Purser J, Hoenig H, Christakis N (2010). Associations of environmental factors with elderly health and mortality in China. Am J Am J Public Health.

[23] Williams BR, Sawyer P, Allman RM (2012). Wearing the garment of widowhood: variations in time since spousal loss among community-dwelling older adults[J]. J Women Aging.

[24] Eibner C, Evans WN (2005). Relative deprivation, poor health habits, and mortality[J]. J Hum Resour.

[25] Callan MJ, Kim H, Gheorghiu AI, Matthews WJ (2017). The interrelations between social class, relative personal deprivation, and prosociality [J]. Social Psychol Personality Sci.

[26] Callan MJ, Shead NW, Olson JM (2015). The relation between relative personal deprivation and the urge to gamble among gamblers is moderated by problem gambling severity: a meta-analysis[J]. Addict Behav.

[27] Mishra S, Carleton RN. Subjective relative deprivation is associated with poorer physical and mental health[J]. Volume 147. Social Science & Medicine; 2015. pp. 144–9. 10.1016/j.socscimed.2015.10.030 26575605

[28] Zhang R, Zhang Y, Xia J. Impact of mobile payment on physical health: evidence from the 2017 China household finance survey[J]. Front Public Health, 2022, 10. 10.3389/fpubh.2022.963234 PMC9376227 35979469

[29] Radloff LS (1991). The Use of the Center for epidemiologic Studies Depression Scale in Adolescents and Young adults [J]. J Youth Adolesc.

[30] Kakwani N (1984). The relative deprivation curve and its applications[J]. J Bus Economic Stat.

[31] Li H, Zhu Y (2006). Income, income inequality, and health: evidence from China[J]. J Comp Econ.

[32] Ling DC (2009). Do the chinese keep up with the Jones? Implications of peer effects, growing economic disparities and relative deprivation on health outcomes among older adults in China[J]. China Econ Rev.

[33] Garbinti B, Goupille-Lebret J, Piketty T (2021). Accounting for wealth-inequality dynamics: methods, estimates, and simulations for France[J]. J Eur Econ Assoc.

[34] Wan G, Wang C, Wu Y (2021). What drove Housing Wealth Inequality in China?. China & World Economy.

[35] Scutella R, Wooden M. The effects of household joblessness on mental health[J]. Volume 67. Social science & medicine; 2008. pp. 88–100. 1. 10.1016/j.socscimed.2008.02.025 18400350

[36] Mullins JT, White C (2019). Temperature and mental health: evidence from the spectrum of mental health outcomes[J]. J Health Econ.

[37] Achim MV, Văidean VL, Borlea SN (2020). Corruption and health outcomes within an economic and cultural framework[J]. Eur J Health Econ.

[38] Belo P, Navarro-Pardo E, Pocinho R et al. Relationship between mental health and the education level in elderly people: mediation of leisure attitude[J]. Front Psychol, 2020, 11. 10.3389/fpsyg.2020.00573 PMC7141236 32296375

[39] Chen Y, Fang H (2021). The long-term consequences of China’s later, longer, fewer campaign in old age[J]. J Dev Econ.

[40] Stuckler D, Basu S, Suhrcke M (2009). The public health effect of economic crises and alternative policy responses in Europe: an empirical analysis[J]. The Lancet.

[41] McInerney M, Mellor JM, Nicholas LH (2013). Recession depression: mental health effects of the 2008 stock market crash[J]. J Health Econ.

[42] Kumar K, Shukla A, Singh A (2016). Association between wealth and health among older adults in rural China and India[J]. J Econ Ageing.

[43] Jou A, Mas N, Vergara-Alert C. Housing wealth, health and deaths of despair[J]. J Real Estate Finance Econ, 2020: 1–34.

[44] Drydakis N. The effect of unemployment on self-reported health and mental health in Greece from 2008 to 2013: a longitudinal study before and during the financial crisis[J]. Volume 128. Social science & medicine; 2015. pp. 43–51. 10.1016/j.socscimed.2014.12.025 25589031

[45] Ribeiro WS, Bauer A, Andrade MCR (2017). Income inequality and mental illness-related morbidity and resilience: a systematic review and meta-analysis[J]. The Lancet Psychiatry.

[46] Matthew P, Brodersen DM (2018). Income inequality and health outcomes in the United States: an empirical analysis[J]. Social Sci J.

[47] Lindqvist E, Östling R, Cesarini D (2020). Long-run effects of lottery wealth on psychological well-being[J]. Rev Econ Stud.

[48] Zimmerman FJ, Bell JF (2006). Income inequality and physical and mental health: testing associations consistent with proposed causal pathways[J]. J Epidemiol Community Health.

[49] He Y, Zhou L, Li J (2021). An empirical analysis of the impact of income inequality and social capital on physical and mental health-take China’s micro-database analysis as an example[J]. Int J Equity Health.

[50] Piera Pi-Sunyer B, Andrews JL, Orben A (2023). The relationship between perceived income inequality, adverse mental health and interpersonal difficulties in UK adolescents[J]. J Child Psychol Psychiatry.

[51] Wilkinson RG (1997). Socioeconomic determinants of health: Health inequalities: relative or absolute material standards?[J]. BMJ.

[52] Belle D, Doucet J (2003). Poverty, inequality, and discrimination as sources of depression among US women[J]. Psychol Women Q.

[53] Siegel AW (2008). Inequality, privacy, and mental health[J]. Int J Law Psychiatry.

[54] Dierckens M, Weinberg D, Huang Y (2020). National-level wealth inequality and socioeconomic inequality in adolescent mental well-being: a time series analysis of 17 countries[J]. J Adolesc Health.

[55] Omer AS, Bezruchka S, Longhi D (2014). The effects of household assets inequality and conflict on population health in Sudan[J]. Afr Popul Stud.

[56] Hong R, Banta JE, Betancourt JA (2006). Relationship between household wealth inequality and chronic childhood under-nutrition in Bangladesh[J]. Int J Equity Health.

[57] He Z, Cheng Z, Bishwajit G (2018). Wealth inequality as a predictor of subjective health, happiness and life satisfaction among nepalese women[J]. Int J Environ Res Public Health.

[58] Alaba O, Chola L (2014). Socioeconomic inequalities in adult obesity prevalence in South Africa: a decomposition analysis[J]. Int J Environ Res Public Health.

[59] Smith ML, Kakuhikire B, Baguma C (2019). Relative wealth, subjective social status, and their associations with depression: cross-sectional population-based study in rural Uganda[J]. SSM-population Health.

[60] Gibson-Davis C, Hill HD (2021). Childhood wealth inequality in the United States: implications for social stratification and well-being[J]. RSF: The Russell Sage Foundation Journal of the Social Sciences.

[61] Marshall A, Jivraj S, Nazroo J (2014). Does the level of wealth inequality within an area influence the prevalence of depression amongst older people?[J]. Health Place.

